# *Lavandula viridis* L´Hér. Essential Oil Inhibits the Inflammatory Response in Macrophages Through Blockade of NF-KB Signaling Cascade

**DOI:** 10.3389/fphar.2021.695911

**Published:** 2022-01-25

**Authors:** Monica Zuzarte, Vera Francisco, Bruno Neves, Joana Liberal, Carlos Cavaleiro, Jorge Canhoto, Lígia Salgueiro, Maria Teresa Cruz

**Affiliations:** ^1^ University of Coimbra, Coimbra Institute for Clinical and Biomedical Research (iCBR), Faculty of Medicine, Coimbra, Portugal; ^2^ University of Coimbra, Center for Innovative Biomedicine and Biotechnology (CIBB), Coimbra, Portugal; ^3^ Clinical Academic Centre of Coimbra (CACC), Coimbra, Portugal; ^4^ Endocrinology and Nutrition Service and Institute of Health Research-INCLIVA, University Clinic Hospital of Valencia, Valencia, Spain; ^5^ Department of Medical Sciences and Institute of Biomedicine (iBiMED), University of Aveiro, Aveiro, Portugal; ^6^ Polytechnic Institute of Castelo Branco, Quality of Life in the Rural World Research Unit (QRural), Castelo Branco, Portugal; ^7^ Faculty of Pharmacy, University of Coimbra, Coimbra, Portugal; ^8^ Department of Chemical Engineering, Faculty of Sciences and Technology, Chemical Process Engineering and Forest Products Research Centre (CIEPQPF), University of Coimbra, Coimbra, Portugal; ^9^ Department of Life Sciences, Faculty of Sciences and Technology, Centre for Functional Ecology (CEF), University of Coimbra, Coimbra, Portugal; ^10^ Centre for Neuroscience and Cell Biology (CNC), Coimbra, Portugal

**Keywords:** COX-2, essentail oil, iNOS, NO, proteasome, ROS, IkB

## Abstract

*Lavandula* *viridis* L´Hér. is an endemic Iberian species with a high essential oil yield and a pleasant lemon scent. Despite these interesting features, this species remains unrecognized and poorly explored by the food and pharmaceutical industries. Nevertheless, it has been valued in traditional medicine being used against flu, circulatory problems and to relieve headaches. Since these disorders trigger inflammatory responses, it is relevant to determine the anti-inflammatory potential of *L. viridis* L´Hér. essential oil in an attempt to validate its traditional use and concomitantly to increment its industrial exploitation. Therefore, in the present study the chemical composition of this volatile extract as well as the effect on ROS production, inflammatory response and proteasome activity on LPS-stimulated macrophages were disclosed. Also, its safety profile on keratinocytes, hepatocytes and alveolar epithelial cells was depicted, envisioning a future human administration. The essential oil was characterized by high quantities of 1,8-cineole, camphor and α-pinene. From a pharmacological point of view, the essential oil showed a potent antioxidant effect and inhibited nitric oxide production through down-modulation of nuclear factor kappa B-dependent *Nos2* transcription and consequently iNOS protein expression as well as a decrease in proteasomal activity. The anti-inflammatory activity was also evidenced by a strong inhibition of LPS-induced *Il1b* and *Il6* transcriptions and downregulation of COX-2 levels. Overall, bioactive safe concentrations of *L. viridis* L´Hér. essential oil were disclosed, thus corroborating the traditional usage of this species and paving the way for the development of plant-based therapies.

## 1 Introduction

The genus *Lavandula* L. comprises a high number of aromatic species with several commercial applications such as a wide diversity of essential oils, fresh and dried flowers as well as landscape plants. The scent of lavender is quite popular in several home, bath care and pet products and provides a unique flavor to beverages, condiments, sweets, marmalades and honey. Moreover, lavender essential oils are a valuable raw material for the food (flavoring), perfumery and cosmetic industries and are widely used in aromatherapy (Boelens, 1985; Upson and Andrews, 2004; Cavanagh and Wilkinson, 2005). Some of these oils are regulated by international ISO standards (ISO, 2007 and 2009), which highlights their high economic value. Despite this popularity, lavenders from the Iberian Peninsula remain poorly explored, thus receiving reduced recognition in global markets.

To fill this gap, the herein study focuses on *L. viridis* L´Hér., a shrub endemic to the south-west Iberian Peninsula. This species presents interesting features such as a high essential oil yield with a lemon scent, very distinct from other lavenders, making it very attractive for industrial exploitation. Nevertheless, *L. viridis* L´Hér. remains undervalued from both a scientific and economic point of view, with only few studies assessing the antifungal (Zuzarte et al., 2011), antioxidant (Matos et al., 2009), nematocidal (Barbosa et al., 2010) and anti-protozoal (Costa et al., 2018; Machado et al., 2019) properties of its essential oils. Therefore, in the present study, the anti-inflammatory potential of the essential oil of *L. viridis* L´Hér. was evaluated to scientifically validate a new bioactivity, thus corroborating its traditional uses and concomitantly adding market value to this underappreciated species. Indeed, *Lavandula viridis* L´Hér. essential oil has been appreciated in aromatherapy due to its sedative and analgesic properties (Cabral et al., 2014) and the plant is traditionally used in infusions to treat flu, circulatory problems and to relieve headaches (Rivera and Obón, 1995). All these disorders trigger inflammatory responses. For instance, viral pathogens, such as influenza, cause pulmonary infections and lung inflammatory responses, associated with inducible nitric oxide synthase (iNOS), cyclooxygenase (COX) and nuclear factor kappa B (NF-kB) activation (Aguilera and Lenz, 2020). Additionally, an exacerbated production of pro-inflammatory mediators was reported in circulatory related disorders, which can still directly activate nociceptors in several pain conditions, like headaches (Matsuda et al., 2019). Therefore, targeting these inflammatory mediators represents an interesting strategy for the treatment of those pathologies. Chronic inflammation is also associated with highly prevalent diseases such as cancer (Coussens and Werb, 2002), obesity, diabetes (Zatterale et al., 2020), rheumatoid arthritis, cardiovascular and neurodegenerative diseases (Hunter and Doddi, 2010) as well as aging. Nowadays, these diseases constitute a major socio-economic burden and novel therapeutic approaches with better safety profiles are highly demanded. Indeed, the commonly used anti-inflammatory drugs such as non-steroidal anti-inflammatory drugs (NSAIDs) are prone to induce severe gastrointestinal and cardiovascular adverse effects. Even biopharmaceuticals such as monoclonal antibodies targeting interleukin-6 (IL-6) or tumor necrosis factor alpha (TNF-α), despite having a safer profile, present several limitations such as the lack of responsiveness and drug resistance, delivery problems and production costs (Awwad and Angkawinitwong, 2018).

From a pathophysiological perspective, tissue inflammatory status is often committed with an overactivation of macrophages responsible for the production of several pro-inflammatory mediators, namely pro-inflammatory cytokines, nitric oxide (NO), prostaglandins (PGs) and reactive oxygen species (ROS) (Medzhitov, 2008). Interestingly, several lines of research have demonstrated the anti-inflammatory activity of essential oils and/or their constituents, pointing out their ability to scavenge free radicals, modulate the arachidonic acid metabolism and the production of cytokines as well as the expression of pro-inflammatory genes (Miguel, 2010; de Lavor et al., 2018).

Regarding the anti-inflammatory potential of *Lavandula* species, the majority of the studies have been carried out in *L. angustifolia* Mill. essential oil. Reports have shown inhibitory effects using the carrageenan-induced paw oedema (Hajhashemi et al., 2003), croton oil-induced ear oedema and dextran-induced paw oedema models (Cardia et al., 2018). Furthermore, the ability of this species to decrease the levels of TNF-α and IL-1β and increase those of IL-10 in a rat model of myocardial infarction has been demonstrated (Souri et al., 2019). Although in less extend, other lavender species also showed anti-inflammatory potential. For instance, the ethanolic fraction of *L. bipinnata* (Roth) Kuntze showed inhibitory effects on COX-2 *in chemico* (Shaikh et al., 2014). The essential oil of *L. luisieri* (Rozeira) Rivas Mart. significantly reduced iNOS and phosphorylated IκB-α in both primary human chondrocytes and in an intestinal cell line (Rufino et al., 2015). *L. stoechas* (Amira et al., 2012) and *L. officinalis* Chaix (= *L. angustifolia* Mill.) (Wei and Shibamoto, 2010) showed strong lipoxygenase inhibitory effects. The ethanolic and aqueous extracts of *L. multifida* L. inhibited croton oil-induced ear oedema in mice (Sosa et al., 2005) and the hydroalcoholic extract of both *L. dentata* L. and *L. stoechas* L. exerted anti-inflammatory effects on a 2,4,6-trinitrobenzene sulfonic acid (TNBS)-induced colitis animal model (Antoniou et al., 2016). Concerning *L. viridis* L´Hér., in a first approach, our group carried out a pilot study demonstrating interesting inhibitory effects on NO production (Zuzarte et al., 2012). Herein, we further explore and deepen the bioactivity and the mechanism of action underlying the observed effects, by assessing the effect of the volatile extract along the canonical inflammatory signaling pathway, the nuclear factor kappa B (NF-κB) pathway, as well as on proteasome activity. To track this goal, an *in vitro* model of inflammation [lipopolysaccharide (LPS)-stimulated macrophages] was selected, thus avoiding animal sacrifices in early stages of drug discovery. The antioxidant potential and the cytotoxicity of the essential oil on several human cell lines were also performed, thus foreseeing possible modes of administration. Altogether, these results show that *L. viridis* L´Hér. essential oil down-modulates the inflammatory response through inhibition of phosphorylation and consequent degradation of IKB-α, thus bringing new insights to the development of plant-based anti-inflammatory strategies and strongly encouraging the industrial valorization of this species.

## 2 Materials and Methods

### 2.1 Plant Material and Isolated Compound

Flowering parts from three representative samples of *L. viridis* L'Hér. (samples A, B and C) collected during Spring in different regions (Barranco do Velho, Salir, Porto Nobre) from Algarve, Portugal were used for essential oil extraction. Voucher specimens were deposited at the Herbarium of the University of Coimbra (COI), with the accession number M. Zuzarte 38. Dr. Jorge Paiva (Department of Life Sciences, University of Coimbra, Portugal), confirmed species authenticity and plant names were checked at http://www.theplantlist.org.

The compound 1,8-cineole was purchased (extra pure, Merck, Darmstadt, Germany).

### 2.2 Isolation and Analysis of Essential Oils

Essential oils from the aerial parts of the plants were obtained by hydrodistillation for 3 h, in a Clevenger-type apparatus, as described by the European Pharmacopoeia (EDQM, 2016). The oils were then preserved in a sealed dark vial at 4°C.

A Hewlett Packard 6890 gas chromatograph (Agilent Technologies, Palo Alto, CA, United States) with a HP GC ChemStation Rev. A.05.04 data handling system, equipped with a single injector and two flame ionization detectors (FID) was used to perform analytical gas chromatography. A graphpak divider (Agilent Technologies, Part Number 5021-7148) allowed simultaneous sampling in two Supelco (Supelco Inc., Bellefont, PA, United States) fused silica capillary columns with distinct stationary phases: SPB-1 (polydimethylsiloxane: 30 m × 0.20 mm i.d., film thickness 0.20 µm) and SupelcoWax 10 (polyethylene glycol: 30 m × 0.20 mm i.d., film thickness 0.20 µm). Oven temperature program: 70–220°C (3°C/min), 220°C (15 min); injector temperature: 250°C; detector carrier gas: He, adjusted to a linear velocity of 30 cm/s; splitting ratio 1:40; detector temperature: 250°C. Gas chromatography-mass spectrometry (GC/MS) analyses were performed on a Hewlett Packard 6890 gas chromatograph fitted with a HP1 fused silica column (polydimethylsiloxane: 30 m × 0.25 mm i.d., film thickness 0.25 µm), interfaced with an Hewlett Packard Mass Selective Detector 5973 (Agilent Technologies, Palo Alto, CA, United States) operated by HP Enhanced ChemStation software, version A.03.00. GC parameters as above; interface temperature: 250°C; MS source temperature: 230°C; MS quadrupole temperature: 150°C; ionization energy: 70 eV; ionization current: 60 μA; scan range: 35–350 μ; scans/s: 4.51.

The compounds were identified by both their retention indices (RI) and their mass spectra. Retention indices were calculated by linear interpolation relative to retention times of a series of n-alkanes and compared with those of authenticated samples from the database of the Laboratory of Pharmacognosy, Faculty of Pharmacy, University of Coimbra. In addition, mass spectra were compared with reference spectra from a home-made library and/or from literature data (Joulain and König, 1998; Adams, 2007). GC peak areas without FID response factor correction were used to calculate relative amounts of the volatile components.

### 2.3 Reagents and Chemicals

Lipopolysaccharide (LPS) from *Escherichia coli* (serotype 026:B6) was purchased at Sigma Chemical Co. (St. Louis, MO, United States). Fetal bovine serum (FBS) and trypsin were obtained from Invitrogen (Paisley, OR, United Kingdom). The protease and phosphatase inhibitor cocktails were obtained from Roche (Mannheim, Germany). Antibodies against phospho-IκB-α and IκB-α, were purchased from Cell Signaling Technologies (Danvers, MA, United States); against iNOS were from R&D Systems (Minneapolis, MN, United States) and against COX-1 and COX-2 from Abcam (Cambridge, United Kingdom). The anti-actin antibody was purchased from Millipore (Bedford, MA). The alkaline phosphatase-linked secondary antibodies and the enhanced chemifluorescence (ECF) reagent were obtained from GE Healthcare (Chalfont St. Giles, United Kingdom). The polyvinylidene difluoride (PVDF) membranes were from Millipore Corporation (Bedford, MA). Trizol^®^ reagent was purchased from Invitrogen (Barcelona, Spain). iScript kit and SYBR green were obtained from BioRad (Hercules, CA, United States). Primers were from MWG Biotech (Ebersberg, Germany). The remaining reagents were from Sigma Chemical Co. (St. Louis, Mo, United States) or from Merck (Darmstadt, Germany).

### 2.4 Cell Lines and Culture Conditions

RAW 264.7 (ATCC - TIB-71), a mouse leukaemic macrophage cell line, was cultured in endotoxin-free Dulbecco’s Modified Eagle Medium (DMEM) (Invitrogen, California, United States) supplemented with 10% (v/v) of non-inactivated FBS, 3.02 g/L sodium bicarbonate, 100 μg/mL streptomycin and 100 U/mL penicillin. HaCat, the human keratinocyte cell line and A549, the human alveolar epithelial cell line (ATCC-CCL-185) were cultured in DMEM medium supplemented with 10% (v/v) of heat inactivated FBS, 3.02 g/L sodium bicarbonate, 100 μg/mL streptomycin and 100 U/mL penicillin. HepG2, an human hepatocyte cell line (ATCC HB-8065) was cultured in DMEM medium supplemented with 10% (v/v) heat inactivated FBS, 1.5 g/L sodium bicarbonate, 100 μg/mL streptomycin and 100U/mL penicillin. Cell lines were maintained at 37°C in a humidified atmosphere of 95% air and 5% CO_2_, and used in the respective experiments after reaching 80–90% confluence. Viable cells were stained with trypan blue dye and counted using a hemocytometer. Their morphological appearance was monitored by optical microscopy during the assays. Cells were regularly sub-cultured and kept in culture for a maximum of 3 months.

### 2.5 Cell Viability

Cell viability was assessed using a colorimetric assay with 3-(4,5-dimethylthiazol-2-yl)-2,5-diphenyl tetrazolium bromide (MTT), as previously described (Mosmann, 1983). Macrophages (3 × 10^5^ cells/well), keratinocytes (2 × 10^5^ cells/well), lung cells (5 × 10^4^ cells/well) and hepatocytes (2 × 10^5^ cells/well) were cultured in 48-well microplates and left to stabilize for 12 h. The cells were further incubated for 24 h with the culture medium alone (control) or with different concentrations of the essential oil (0.08–1.25 μL/mL) or 1,8-cineole (0.16–2.5 μL/mL, for macrophages). Then, a MTT solution (final concentration 0.5 mg/mL) was added and cell cultures incubated at 37°C for additional 15 min (macrophages), 30 min (keratinocytes), 2.5 h (alveolar epithelial cells) or 1 h (hepatocytes), in a humidified atmosphere of 95% air and 5% CO_2_. Supernatants were centrifuged at 1,000 g during 5 min to recover viable cells. Formazan crystals formed in adherent cells were dissolved using 300 µL of acidified isopropanol (0.04 N HCl in isopropanol) and recovered to the respective microtube containing the pellet. Quantification was performed on an ELISA automatic microplate reader (SLT, Austria) at 570 nm, with a wavelength of 620 nm. A cell-free control was performed in order to exclude non-specific effects of the essential oil/compound on MTT (data not shown). All the experiments were performed in triplicate and results expressed as mean ± SEM of at least three independent experiments.

### 2.6 Antioxidant Activity

#### 2.6.1 Nitric Oxide Scavenging Potential

To assess the NO scavenging potential of the essential oil, S-nitroso-N-acetyl-DL-penicillamine (SNAP) was used as a NO donor. First, 300 µL of culture medium alone (control) or with different concentrations of the essential oil (0.08–0.64 μL/mL) and SNAP (300 µM) were incubated in 48-well microplates, for 3 h at 37°C. Then, 170 µL of the supernatants were mixed with 170 µL of Griess reagent (1% sulphanilamide and 0.1% naphthylethylenediamine dihydrochloride in 2.5% phosphoric acid) and incubated for 30 min, in the dark, at room temperature. A microplate reader was used to register the absorbance at 550 nm and a sodium nitrite standard curve used to determine the amount of nitrites. All experiments were performed in triplicate.

#### 2.6.2 Reactive Oxygen Species Production

RAW 264.7 cells (5 × 10^4^ cells/well) were plated in a µ-Chamber slide (IBIDI GmbH, Germany), allowed to stabilize overnight and then treated with LPS (1 μg/mL) during 16 h. When indicated, *L. viridis* L´Hér. essential oil (0.64 μL/mL) was added 1 h prior to LPS. At the end of the incubation period, cells were washed three times and then loaded with 5 μM H2DCFDA (fluorescent indicator of ROS) and 0.5 μg/mL Hoechst (DNA stain) in HBSS for 30 min at 37°C in the dark. Cells were washed with HBSS and analysed using an Zeiss Axio Observer.Z1 inverted microscope (Carl Zeiss, Germany) equipped with an AxioCam HRm and Zen Blue 2012 software, using a 63× oil objective (Plan-Apochromat, 1.4 NA).

### 2.7 Anti-inflammatory Activity

#### 2.7.1 Nitric Oxide Production

The accumulation of nitrites (a stable metabolite of NO) was measured in the culture medium by a colorimetric assay using Griess reagent (prepared as referred in 2.6.1). The cells (3 × 10^5^ cells/well) were cultured in 48-well microplates and left to stabilize for 12 h. The cells were then incubated for 24 h in the culture medium alone (control) or with different concentrations of the essential oil (0.08–0.64 μL/mL) or 1,8-cineole (0.16–2.5 μL/mL) and stimulated with LPS (1 μg/mL). Following treatments, 170 µL of the supernatants were mixed with 170 µL of Griess reagent and incubated in the dark for 30 min, at room temperature. The absorbance was read at 550 nm in a microplate reader. The quantity of nitrites was determined based on a sodium nitrite standard curve. All experiments were performed in triplicate.

#### 2.7.2 Protein Expression

Macrophages were plated (6 × 10^5^ cells/well), allowed to stabilize for 12 h and then either maintained in culture medium (control) or pre-incubated with the essential oil (0.64 μL/mL) for 1 h followed by LPS during 24 h or the time indicated in the results section. Also, incubation of the oil with LPS at the same time was assessed. To obtain total cellular lysates, lysis buffer (RIPA: 50 mM Tris-HCL, pH 8.0, 1% Nonidet P-40, 150 mM NaCl, 0.5% sodium deoxycholate, 0.1% sodium dodecyl sulphate and 2 mM ethylenediaminetetraacetic acid) freshly supplemented with 1 mM dithiothreitol, protease and phosphatase inhibitor cocktails was used. Then, cell lysates were sonicated (4x, 40 µm peak to peak) in a Vibra Cell sonicator (Sonica and Material INC) and centrifuged for 10 min at 4°C to remove nuclei and cell debris. Supernatants (total cell lysates) were collected and protein concentration determined by the bicinchoninic acid protein assay. Then, cell lysates were denatured in sample buffer (0.125 mM Tris pH 6.8, 2% (w/v) sodium dodecyl sulphate, 100 mM dithiothreitol, 10% glycerol and bromophenol blue) and Western blot analysis was carried out to assess protein levels of pIKB-α, IKB-α, iNOS and COX-2. In summary, equal amounts of proteins were separated on a 10% (v/v) sodium dodecylsulphate-polycrylamide gel at 140 v, for 1h, and then transferred to PDVF membranes. Nonspecific IgGs were blocked with 5% (w/v) milk in Tris-buffered saline (TBS, 50 mM Tris-HCL, pH 7.6, 150 mM NaCl) and incubated at room temperature, for 1 h. Then, membranes were incubated with specific antibodies against iNOS (1:1,000 dilution), COX-2 (1:10,000), total IκBα (1:1,000), phospho-IκB (1:1,000) or actin (1:20,000) overnight at 4°C. The membranes were then washed with TBS-T (total 25 min) and incubated, for 1 h, at room temperature, with alkaline phosphatase-conjugated secondary antibodies. The immune complexes were detected by membrane exposure to the ECF reagent (GE Healthcare) during 5 min, using the imaging system Typhoon TM FLA 9000 and generated signals analyzed using the ImageQuant TL software.

#### 2.7.3 RNA Extraction and Real-Time PCR

Cells were plated at 4 × 10^4^ cells/well in 6-well microplates in a final volume of 2 mL. Then, cells were either maintained in culture medium (control) or pre-incubated with the essential oil (0.64 μL/mL) for 1 h followed by LPS (1 μg/mL) during 6 h.

Total RNA was isolated using Trizol^®^ reagent according to the manufacturer’s instructions. Briefly, cells were washed with ice-cold PBS and then harvested and homogenized in 1 mL of Trizol. After the addition of 200 μL of chloroform, the samples were vortexed, allowed to incubate for 2 min and centrifuged at 12,000 g, for 15 min, at 4°C. The aqueous phase (RNA) was transferred to a new tube and RNA precipitated with 500 µL of isopropanol for at least 10 min. Following a 10 min centrifugation at 12,000g, the pellet was washed with 1 mL 75% ethanol and resuspended in 100 μL 60°C heated RNAse free water. RNA concentration was determined by OD260 measurement using a Nanodrop spectrophotometer (Wilming-ton, DE, United States) and quality was inspected for absence of degradation or genomic DNA contamination, using the Experion RNA StdSens Chips in the ExperionTM automated microfluidic electrophoresis system (BioRad Hercules, CA, United States). RNA was stored in RNA Storage Solution (Ambion, Foster City, CA, United States) at −80°C.

For real-time PCR, 1 µg of total RNA was reverse transcribed using the iScript Select cDNA Synthesis Kit. Briefly, 2 µL of random primers and the necessary volume of RNase-free water to complete 15 μL, were added to each RNA sample. The samples were heated at 65°C, for 5 min, and snap-chilled on ice for 1 min. After this, 5 µL of a Master Mix containing 1 µL of iScript reverse transcriptase and 4 µL of 5x Reaction Buffer were added to each sample. A protocol for cDNA synthesis was run on all samples (5 min at 25°C, 30 min at 42°C, 5 min at 85°C and then put on hold at 4°C). Following cDNA synthesis, samples were diluted with RNase-free water up to 100 µL. Real-time PCR was carried out in a 20 µL volume [50 ng 5 µL cDNA, 10 µL 2x Syber Green Supermix, 2 µL of each primer (250 nM) and 1 µL H_2_O PCR grade]. Samples were denatured at 95°C during 3 min and then 40 cycles were run for 10 s at 95°C for denaturation, 30 s at respective annealing temperature and 30 s at 72°C for elongation. Real-time PCR reactions were carried out on a Bio-Rad My Cycler iQ5, in duplicate. Primers (Table 1) were designed using Beacon Designer^®^ Software v7.2 (Primier Biosoft International).

**TABLE 1:** Primer sequences for targeted cDNAs.

Primer	5′-3′ sequence (F: forward; R: Reverse)	RefSeq ID
*Hprt1*	F: GTT​GAA​GAT​ATA​ATT​GAC​ACT​G	NM_013556
	R: GGC​ATA​TCC​AAC​AAC​AAA​C	
*Il1b*	F: ACC​TGT​CCT​GTG​TAA​TGA​AAG	NM_008361
	R: GCTTGTGCTCTGCTTGTG	
*Il6*	F: TTCCATCCAGTTGCCTTC	NM_031168
	R: TTC​TCA​TTT​CCA​CGA​TTT​CC	
*Nos2*	F: GCT​GTT​AGA​GAC​ACT​TCT​GAG	NM_000625.4
	R: CAC​TTT​GGT​AGG​ATT​TGA​CTT​TG	

A non-template control was included for each pair of primers. In addition, dilution series of control sample for each pair of primers was used to determine primer-pair specific efficiencies. After amplification, a threshold was set for each gene and Ct-values were calculated. Gene expression changes were analysed using the built-in iQ5 Optical system software v2 though the Pfaffl method (Pfaffl, 2001). Results were normalized using *HPRT-1* as a reference gene previously determined with Genex^®^ software (MultiD Analyses AB).

### 2.8 Statistical Analyses

Results are expressed as mean ± SEM of at least three independent experiments. Statistical analysis comparing a treatment condition to control was performed using two-sided unpaired *t*-test. To compare the effect of different treatments to control or LPS-stimulated cells, one-way or two-way ANOVA followed by Dunnett’s multiple comparison test with significance levels (*p < 0.05, **p < 0.01, ***p < 0.001, ****p < 0.0001) was used. These tests were applied using GraphPadPrism version 9 (GraphPad Software, San Diego, CA, United States). For PCR results, a two-base logarithmic transformation was used to make observations symmetric and closer to a normal distribution. If x represent the fold change of a gene in one sample, then the two-base logarithmic transformation is: log2 (x) =ln (x1)/ln (2). In this way, fold changes of 2 and 0.5 correspond to mean log2 values of 1 and −1, respectively.

## 3 Results

### 3.1 Essential Oil Characterization

The chemical composition of three representative essential oils (samples A, B and C) from *L. viridis* L´Hér. collected at different regions of south Portugal (Barranco do Velho, Salir and Porto Nobre, respectively) is listed in Table 2. The essential oils showed a yield varying from 1.5–1.8% (v/w) which makes this species very interesting under an industrial point of view. Overall, fifty-one compounds were identified, representing more than 90% of the total volatile extracts. The oils were characterized by high amounts of oxygen-containing monoterpenes (70.5–76.3%), followed by monoterpenic hydrocarbons (15.5–21.8%), being the main constituents 1,8-cineole (34.5–42.2%), camphor (12.8–13.4%) and α-pinene (9.0–14.1%). Since *L. viridis* L´Hér. essential oils showed a high homogeneity among samples, only the most representative one, sample (A), was selected to carry out the following experiments.

**TABLE 2:** Composition (%) of the essential oils of *Lavandula viridis* from three regions of Portugal.

RI	RI	Compound	Barranco do Velho (A)	Salir (B)	Porto Nobre (C)
SPB-1	SW 10				
920	1020	tricyclene	0.3	0.3	0.4
**930**	**1030**	**α-pinene**	**9.0**	**9.0**	**14.1**
942	1075	camphene	3.3	3.7	4.3
942	1129	verbenene	0.3	0.4	0.7
951	1303	1-octen-3-one	1.0	1.4	2.0
960	1337	hept-5-ene-6-methyl-2-one	0.2	0.3	0.1
963	1253	3-octanone	0.1	0.2	0.1
964	1127	sabinene	0.3	0.1	0.1
969	1116	β-pinene	1.1	0.5	0.5
977	1388	3-octanol	0.2	t	t
980	1161	myrcene	0.2	0.1	0.1
1008	1185	α-terpinene	0.2	0.1	0.2
1012	1272	*p*-cymene	0.3	0.1	0.1
**1020**	**1215**	**1,8-cineole**	**34.5**	**42.2**	**40.5**
1021	1205	limonene	0.8	0.6	0.5
1027	1233	*cis*-β*-*ocimene	0.7	0.4	0.6
1035	1249	*trans-*β*-*ocimene	0.4	0.1	0.1
1047	1249	γ-terpinene	0.2	0.1	0.1
1051	1459	*trans*-sabinene hydrate	0.2	0.1	0.1
1055	1439	*cis*-linalool oxide	0.9	1.2	0.7
1070	1467	*trans*-linalool oxide	1.0	1.0	0.6
1082	1542	linalool	7.9	6.7	6.0
1098	1371	oct-1-en-3-yl acetate	0.2	0.2	0.2
1102	1489	α-campholenal	0.3	0.4	0.4
1104	1574	nopinone	0.2	0.2	0.2
**1118**	**1514**	**camphor**	**13.4**	**13.4**	**12.8**
1121	1645	*cis*-verbenol	0.8	0.5	0.6
1125	1669	*trans*-verbenol	1.0	0.8	1.0
1134	1563	pinocarvone	0.4	0.3	0.4
1143	1721	*p*-mentha-1,5-dien-8-ol	1.2	1.2	1.0
1146	1663	isoborneol	0.6	0.5	0.4
1146	1692	borneol	2.8	3.1	3.1
1158	1595	terpinen-4-ol	0.9	1.0	0.6
1165	1621	myrtenal	0.4	0.2	0.1
1169	1689	α-terpineol	0.9	1.2	0.7
1176	1694	verbenone	1.3	0.9	1.0
1196	1829	*trans*-carveol	0.3	0.4	0.4
1212	1728	carvone	0.2	0.2	0.2
1237	1553	linalyl acetate	0.2	0.1	0.1
1264	1574	bornyl acetate	0.2	0.2	0.2
1342	1459	α-cubebene	t	t	t
1358	1740	myrtenyl acetate	0.2	0.1	0.3
1359	1750	geranyl acetate	0.7	0.4	0.2
1468	1688	γ-curcumene	0.7	0.6	0.2
1470	1716	β-salinene	0.3	0.1	0.1
1494	1747	γ-cadinene	1.1	0.9	0.4
1521	1773	γ-selinene	0.5	0.1	0.1
1529	1773	selina-3,7(11)-diene	0.9	0.2	0.1
1529	1769	α-bisabolene	0.6	0.1	0.1
1628	2,218	α-cadinol	0.3	0.1	0.1
1667	1709	β-bisabolol	0.3	0.1	0.1
Monoterpene hydrocarbons			17.1	15.5	21.8
Oxygen containing monoterpenes			70.5	76.3	71.6
Sesquiterpene hydrocarbons			4.1	2.0	1.0
Oxygen containing sesquiterpenes			0.6	0.2	0.2
Others			1.7	2.1	2.4
**Total identified**			**94.0**	**96.1**	**97.0**

Compounds listed in order of elution from the SPB-1 column.

Compounds highlighted in bold are the main compounds of the essential oils.

RI SPB-1: GC-retention indices relative to C9–C23 n-alkanes on the SPB-1 column.

RI SW 10: GC-retention indices relative to C9–C23 n-alkanes on the SupelcoWax-10 column.

t= traces (≤0.05%).

### 3.2 Effect of *Lavandula viridis* L´Hér. Essential Oil on Cell Viability

**FIGURE 1:**
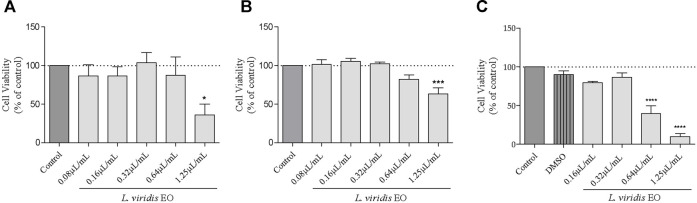
Effect of *L. viridis* L´Hér. essential oil (EO) on human cell lines viability: **(A)** HepG2, **(B)** A549 and **(C)** HaCAT. Cells were treated with different concentrations of the essential oil for 24 h **(A, B, C)** or DMSO **(C)**. Cell viability is expressed as a percentage of MTT reduction in comparison to control cells (100% viability). Each value represents the mean ± SEM from three experiments, performed in duplicate. Statistical differences between groups were calculated by one-way ANOVA followed by Dunnet’s post hoc test (*p < 0.05, ***p < 0.001, ****p < 0.0001, compared to control).

The effect of the essential oil was assessed using a cell viability assay (MTT) in order to disclose safe concentrations. Different human cell lines were selected to mimic an oral (hepatocytes—HepG2), through inhalation (epithelial lung cells–A549) or topical (keratinocytes–HaCAT) oil administration. For both hepatocytes (Figure 1A) and epithelial alveolar cells (Figure 1B), the essential oil affected cell viability only at the higher concentration tested (1.25 μL/mL), whereas in keratinocytes the oil was slightly more toxic, with cell viability being affected at 0.64 μL/mL (Figure 1C).

### 3.3 Effect of *Lavandula viridis* L´Hér. Essential Oil on Nitric Oxide Production

**FIGURE 2:**
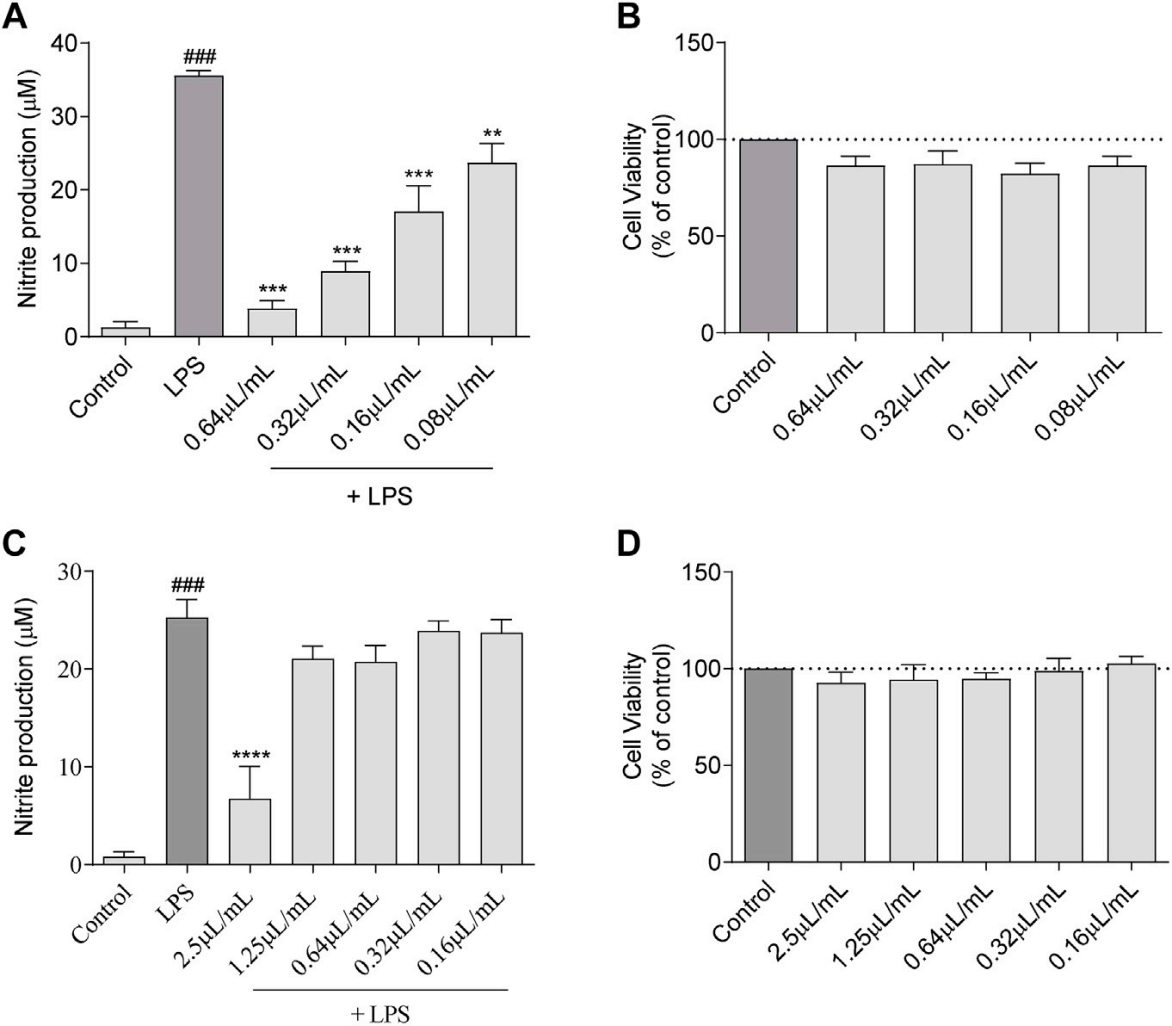
NO production: **(A)** Effect of *L. viridis* L´Hér. essential oil (EO) on nitrite production. **(B)** Effect of *L. viridis* L´Hér. essential oil on macrophages viability. **(C)** Effect of 1,8-cineole on nitrite production. **(D)** Effect of 1,8-cineole on macrophages viability. Cell viability is expressed as a percentage of MTT reduction in comparison to control cells (100% viability). Each value represents the mean ± SEM from at least three experiments, performed in duplicate. Statistical differences between control and LPS groups were calculated using two-sided unpaired *t*-test and differences between a treatment condition and LPS was performed using one-way ANOVA followed by Dunnet’s post hoc test (###*p* < 0.001, compared to control; **p < 0.01, ***p < 0.001, ****p < 0.0001 compared to LPS).

As LPS induces an increase in nitrites (a stable metabolite of NO) production in macrophages, thus mimicking an inflammatory response in the body, it may be used to screen the anti-inflammatory potential of extracts/compounds. Herein, untreated Raw 264.7 cells produced very low levels of nitrites (1.31 µM) but following stimulation with LPS these levels increased to 35.57 µM (Figure 2A). In cells pre-incubated with *L. viridis* L´Hér. essential oil, this production was strongly inhibited in a dose-dependent way (Figure 2A). Indeed, all the tested concentrations were able to significantly inhibit nitrite production and at the higher concentration tested (0.64 μL/mL) a striking inhibition of 96.15% was achieved without affecting cell viability (Figure 2B), thus pointing out a promising anti-inflammatory potential of the volatile extract.

The major compound of *L. viridis* L´Hér. essential oil, 1,8-cineole, was also assessed in order to disclose whether this compound is responsible for the anti-inflammatory effect of the volatile extract. Nevertheless, in comparison to the essential oil, a much higher concentration of 1,8-cineole is required to significantly reduce nitrite production (Figures 2C,D), thus suggesting that other compounds may also be responsible for the activity of the oil or that synergistic effects between compounds may occur.

### 3.4 Antioxidant Effect of *Lavandula viridis* L´Hér. Essential Oil

**FIGURE 3:**
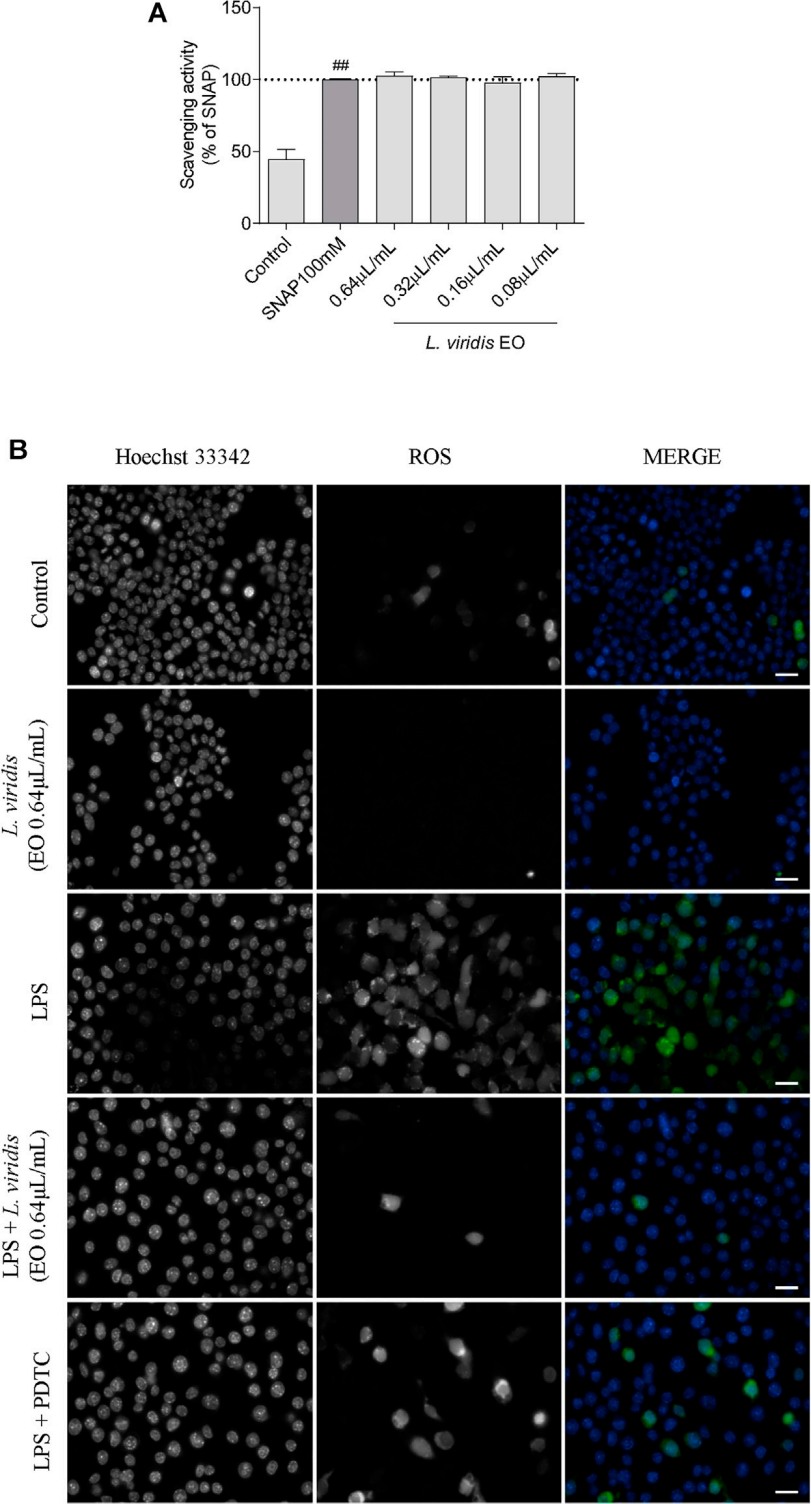
Antioxidant potential of *L. viridis* L´Hér. essential oil (EO): **(A)** Effect on reactive nitrogen species (RNS) assessed though a nitrite-scavenging assay. Different concentrations of the essential oil were incubated with the NO donor, SNAP (100 mM), in culture medium for 3 h. Results are expressed as a percentage of NO release by SNAP. Each value represents the mean ± SEM from three experiments, performed in duplicate. Statistical differences between control and SNAP groups were calculated using two-sided unpaired *t*-test (###*p* < 0.001, compared to control). **(B)** Immunofluorescence analysis of the effect of *L. viridis* L´Hér. on reactive oxygen species (ROS) production. Macrophages were incubated on μ-slides and treated as detailed in the methods section. PDTC was used as a positive control and Hoechst 33342 used as counter stain. The images were acquired with an Axio Observer.Z1 inverted fluorescence microscope (Zeiss). Scale bar: 20 µm.

The antioxidant potential of the essential oil towards reactive nitrogen species (RNS) and reactive oxygen species (ROS) was assessed. In the first case, using an *in vitro* nitrite-scavenging assay, the results obtained demonstrated that the essential oil was ineffective with no NO scavenging effect observed at the tested concentrations (Figure 3A). Indeed, the recorded values of nitrites remained very similar to the NO donor, SNAP. In what concerns ROS, representative images of fluorescence microscopy (Figure 3B) show that the inflammatory stimuli, LPS, induced a substantial increase in ROS production. Interestingly, in the presence of the essential oil (0.64 μL/mL), ROS production was abolished attaining similar levels to those observed in the control. Importantly, this effect was more robust than that observed for the antioxidant pyrrolidine dithiocarbamate (PDTC), thus reinforcing the strong antioxidant potential of *L. viridis* L´Hér. essential oil towards inflammation-induced ROS (Figure 3B). This preventive effect over LPS-induced ROS was also observed for lower doses of the essential oil as shown in Supplementary Material (Supplementary Figure S1).

### 3.5 Effect of *Lavandula viridis* L´Hér. Essential Oil on NF-κB Signaling Pathway

Self-limiting acute inflammation is generally attenuated after elimination of the harmful stimuli, leading to restoration of homeostasis and initiation of tissue repair. However, unresolved inflammation may promote the development of chronic auto-immune and degenerative diseases as well as cancer. One of the key molecular mechanism that contributes to the perpetuation of chronic inflammation is the activation of NF-κB signaling cascade, which has emerged as the master regulator of inflammation and innate immune homeostasis. Therefore, we evaluated the anti-inflammatory potential of the essential oil by studying its effect on the activation of NF-κB signaling pathway. Several key points along this pathway were considered, namely the effect of the essential oil on the proteolytic degradation of the inhibitor (IκB-α), on proteasome activity and at transcriptional and translational levels of pro-inflammatory molecules dependent on NF-κB activation, as depicted next.

#### 3.5.1 Effect on the Proteolytic Degradation of IκB-α

**FIGURE 4:**
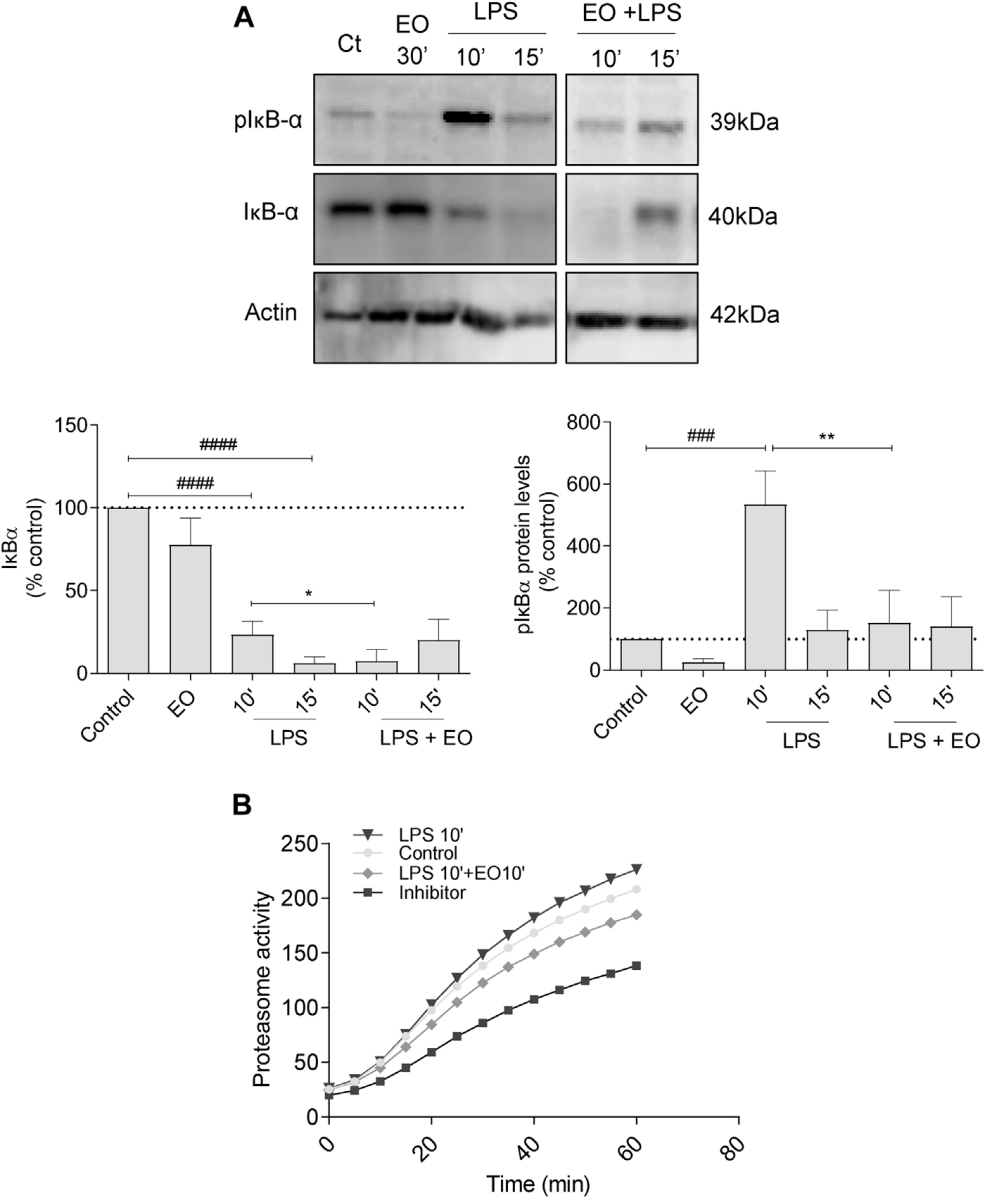
Effect of *L. viridis* L´Hér. essential oil (EO) on the proteolytic degradation of IκB-α: **(A)** Protein expression of pIκB-α and IκB-α. Raw 264.7 cells were maintained in culture medium (control), or incubated with LPS, or the EO (0.64 μL/mL) alone for 30 min, or simultaneously with LPS and the EO for 10 or 15 min. **(B)** Effect of *L. viridis* L´Hér. essential oil on proteasome activity. Raw 264.7 cells were maintained in culture medium (control), or incubated with proteasome inhibitor MG-132, or the EO added 10 min prior to reading. Each value represents the mean ± SEM from at least three experiments, performed in duplicate. Statistical differences between control and LPS groups were calculated using two-sided unpaired *t*-test and differences between a treatment condition and LPS alone was performed using one-way ANOVA followed by Dunnet’s post hoc test (###*p* < 0.001, ####*p* < 0.0001, compared to control; *p < 0.05, **p < 0.01, compared to LPS 10 min).

Incubation of the cells with LPS increased the phosphorylation of the inhibitor of NF-κB, IκB-α (mainly after 10 min of stimulation), with concomitant ubiquitination and proteasomal degradation. This is evident by the low IκB-α levels observed at 15 min post LPS treatment. Notably, *L. viridis* L´Hér. essential oil significantly inhibited LPS-triggered IκB-α phosphorylation and consequent proteasomal targeting, thus compromising the activation of NF-κB signaling pathway (Figure 4A). In addition, the essential oil showed a tendency to decrease proteasome activity (Figure 4B), thus strengthening its inhibitory effect on NF-κB signaling pathway activation.

#### 3.5.2 Effect on the mRNA Levels of *Nos2*, *Il1b* and *Il6*

**FIGURE 5:**
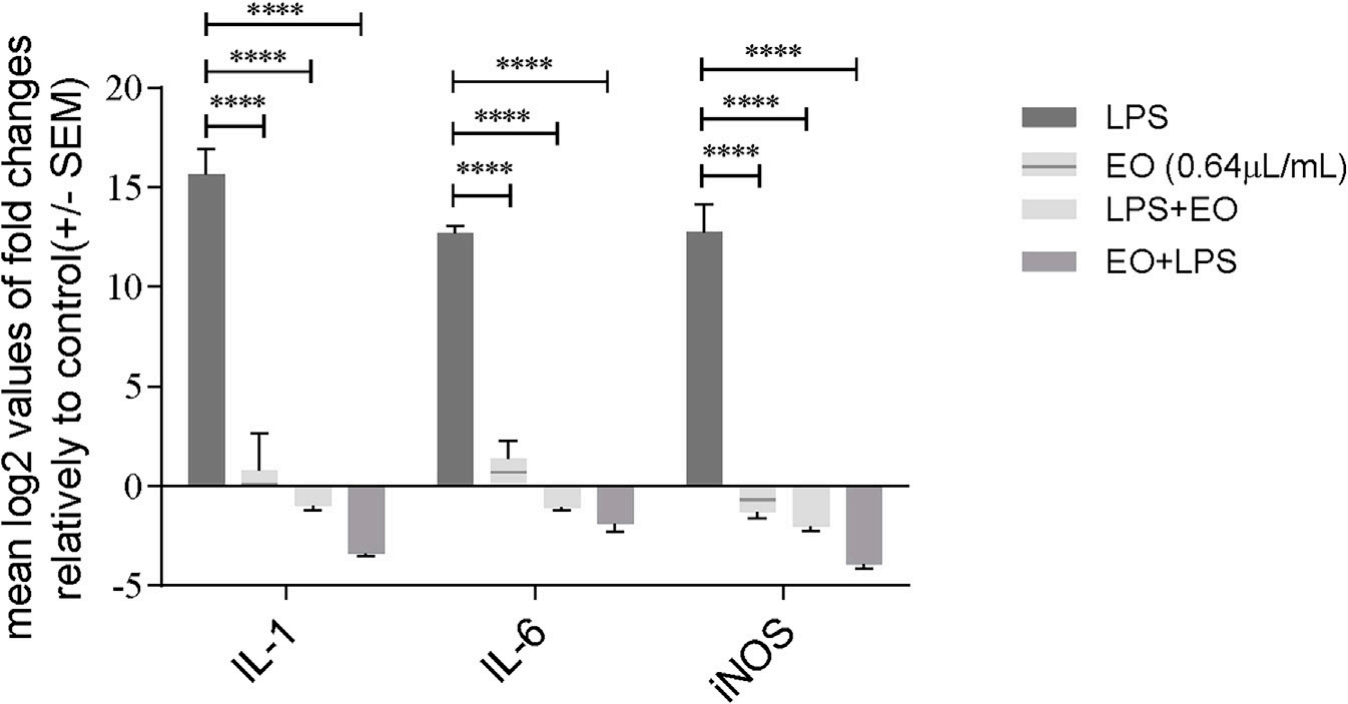
Modulation of LPS-induced transcription of pro-inflammatory genes *Il1b*, *Il6* and *Nos2* by *L. viridis* L´Hér. essential oil added simultaneously or 1 h prior to LPS. Cells were treated with LPS during 6 h. The mRNA levels were assessed by quantitative Real-Time RT-PCR. Gene expression is indicated as mean log2 values of fold changes relatively to control and normalized to *HPRT1* housekeeping gene. Each value represents the mean ± SEM. from three independent experiments. Statistical differences between groups were calculated by two-way ANOVA followed by Dunnet’s post hoc test (****p < 0.0001, compared to LPS).

Based on the previous results, we hypothesized that the inhibition of IκB-α phosphorylation and degradation would down-regulate the transcription of NF-κB target genes. To confirm this rationale, we assessed the effect of the essential oil on the mRNA levels of both inducible nitric oxide synthase (*Nos2*) and the pro-inflammatory cytokines interleukins *Il1b* and *Il6*. The essential oil alone did not induce the transcription of the studied genes, thus confirming the absence of both a pro-inflammatory effect and eventual endotoxin contamination (Figure 5). On the other hand, LPS treatment increased the mRNA levels of both *Nos2* and pro-inflammatory cytokines, being this effect significantly abrogated in the presence of the essential oil (Figure 5). A most significant inhibition was observed for *Il1b* and *Nos2* when the essential oil was pre-incubated for 1 h before LPS stimulation (Figure 5). However, significant decreases were also observed when the essential oil was added after LPS stimulation, indicating that it is able to revert the pro-inflammatory status elicited by LPS (Figure 5).

#### 3.5.3 Effect on iNOS and COX-2 Protein Levels

**FIGURE 6:**
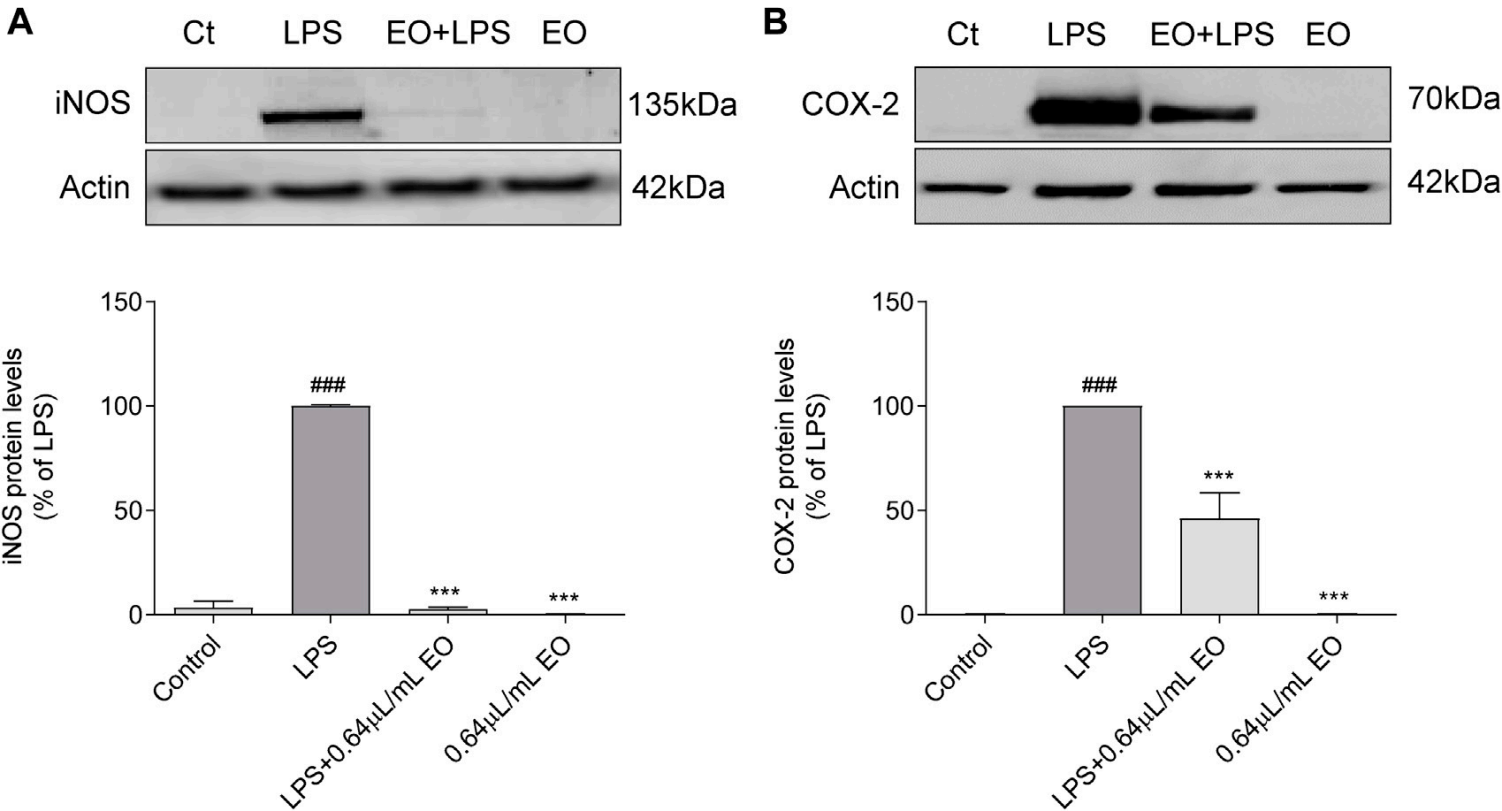
Inhibitory effect of *L. viridis* L´Hér. essential oil (EO) on LPS-induced: **(A)** iNOS protein expression and **(B)** COX-2 protein expression in macrophages. Raw 264.7 cells were maintained in culture medium (control), or incubated with LPS, or with the essential oil (0.64 μL/mL) alone or simultaneously with LPS for 24 h. Results are expressed as percentage of iNOS or COX-2 protein levels relatively to LPS. Each value represents the mean ± SEM from at least three experiments. Statistical differences between control and LPS groups were calculated using two-sided unpaired *t*-test and differences between a treatment condition and LPS alone was performed using one-way ANOVA followed by Dunnet’s *post hoc* test (###*p* < 0.001, compared to control; ***p < 0.001, compared to LPS).

The effect of *L. viridis* L´Hér. essential oil on LPS-induced iNOS (Figure 6A) and COX-2 (Figure 6B) protein levels, was evaluated by western blot. In untreated cells (control) and in cells treated with the essential oil alone, no iNOS (Figure 6A) or COX-2 (Figure 6B) proteins were detected. However, after macrophages stimulation with LPS during 24 h, the expression of both enzymes was strongly increased (Figures 6A,B, respectively). Pre-treatment of cells with the essential oil significantly reduced the LPS induced iNOS expression by 97.40% (Figure 6A). Regarding COX-2 expression, a decrease of 53.9% was also attained (Figure 6B). These results are quite remarkable since blockade of COX-2 protein expression by natural products is not frequent. In addition, the essential oil seems to selectivity inhibit COX-2 expression, since no effects were observed on COX-1 levels (Supplementary material–Supplementary Figure S2), thus strengthening its relevance as a potential COX-2 selective anti-inflammatory drug and therefore likely presenting less adverse effects on the gastric mucosa.

## 4 Discussion

In the present study the essential oil of different samples of *L. viridis* L´Hér. was isolated and characterized. All samples showed a similar chemical profile, with 1,8-cineole, camphor and α-pinene being the main compounds. Previously, Garcia Vallejo (Garcia Vallejo, 1992) analyzed individual samples of *L. viridis* L´Hér. from the south of Portugal and Spain. The chemical composition reported was very similar to that of our collective samples, with 1,8-cineol being the major component in all samples. These findings point to a very high chemical homogeneity in the essential oils of *L. viridis* L´Hér. from both Portugal and Spain, a very relevant feature for industrial use. In addition, *L. viridis* L´Hér. presents a high essential oil yield and a pleasant lemon scent that are also interesting features for an industrial application.

Although several essential oils from *Lavandula* spp. have been assessed for their antioxidant and anti-inflammatory potential (Miguel, 2010; de Lavor et al., 2018), most of the studies focus on *L. angustifolia* Mill. Since *L. viridis* L´Hér. remains undervalued, we sought to increment its industrial relevance by assessing the antioxidant and anti-inflammatory potential of its volatile extract. The present study clearly demonstrated a potent effect on ROS inhibition as well as a strong anti-inflammatory potential. The later was, at least in part, due to the modulation of the pro-inflammatory signaling cascade NF-κB. Indeed, the inflammatory response is a well-coordinated and controlled balance of particular intracellular pathways, namely the NF-kB signaling cascade. The activation of this cascade leads to the induction of inflammation-related proteins, such as iNOS and COX-2, as well as several inflammatory mediators like TNF-α, IL-6 and IL-1(Edwards et al., 2009; Wong and Tergaonkar, 2009). NF-κB can be activated by inflammatory stimuli like LPS, cytokines and oxidants through several signaling pathways that converge to the phosphorylation of its inhibitor (IkB) leading to its ubiquitination and subsequent degradation by the proteasome. IkB degradation unmasks the nuclear localization motif of NF-κB, allowing its rapid translocation to the nucleus and subsequent transcription of target genes. Due to the importance of this pathway in the modulation of the inflammatory response, NF-κB inhibition is an important target in the development of new anti-inflammatory drugs. Accordingly, we demonstrated that *L. viridis* L´Hér. essential oil interferes with the NF-κB signaling pathway at several levels. It impairs the phosphorylation of IkB-α consequently preventing its ubiquitination and proteasomal targeting. Additionally, the essential oil directly decreased proteasomal activity. The conjunction of these two effects culminates in an impaired degradation of IkB-α and consequent decrease of NF-κB translocation to the nucleus. This inhibition of NF-κB signaling cascade justifies the observed inhibition of LPS induced transcription of *Il1b*, *Il6* and *Nos2* and induced expression of COX-2 and iNOS proteins. The reduction of iNOS expression was responsible for the decrease in nitrites levels after macrophages stimulation with LPS. Hence, considering the results obtained, it seems reasonable that the inhibitory effects of *L. viridis* L´Hér. essential oil on the production of NO occurs via modulation of the NF-κB signaling cascade. However, it must be stated that the inhibition of other transcription factors can also occur, thus contributing to the anti-inflammatory profile of the volatile extract.

Considering that 1,8-cineol is the main compound of *L. viridis* L´Hér. essential oil, we also assessed its effect. Our results demonstrated that 1,8-cineole was able to inhibit NO production but at a higher concentration than that used for the essential oil, suggesting that other compounds are also responsible for the anti-inflammatory profile of the oil. Indeed, as essential oils are complex mixtures of several compounds, their activity may be due to the contribution of several compounds in addition to their major compound. In fact, the anti-inflammatory effects of other important compounds found in *L. viridis* L´Hér. essential oil, namely α-pinene and camphor, have been reported. For example, α-pinene was able to attenuate neuro-inflammation in a rat model of focal cerebral ischemia-reperfusion by decreasing both the gene and protein expression of TNF-α and IL-1β (Khoshnazar et al., 2020). Moreover, in mouse peritoneal macrophages, this compound showed anti-inflammatory potential by suppressing the mitogen-activated protein kinases (MAPKs) and the NF-κB pathways (Kim et al., 2015). Furthermore, a selective and more potent anti-inflammatory effect was shown in human chondrocytes, for the (+)-α-pinene in comparison to the (-)-enantiomer, through inhibition of the IL-1β-induced inflammatory and catabolic pathways, namely, NF-κB and c-Jun N-terminal kinase (JNK) activation (Rufino et al., 2014). Regarding camphor, dos Santos and colleagues reported anti-inflammatory articular effects in zymosan induced-articular inflammation in mice. The authors showed that the increase in all articular parameters induced by zymosan, namely knee edema, leukocyte infiltration, mechanical hyperalgesia and NO, were prevented with camphor administration (dos Santos et al., 2021). In addition, synergistic effects between essential oil compounds are frequent and minor compounds may contribute to the overall observed effect.

The conventional anti-inflammatory drugs dexamathasone (Francisco et al., 2011) and indomethacine (Tavares et al., 2015) were previously assessed in our laboratory. Interestingly, a much stronger inhibition in both, NO production and iNOS expression was observed with *L. viridis* L´Hér. essential oil in comparison to these drugs, without affecting cell viability. Furthermore, the oil was also able to selectively inhibit the expression of COX-2 (inducible form), without interfering with that of COX-1 (constitutive and cytoprotective form). Indeed, bioactive and safe concentrations of *L. viridis* L´Hér. essential oil were disclosed using different human cell lines, that were selected bearing in mind possible routes of administration. Although cell viability results require additional consideration when transported to an *in vivo* setting, they are well suited for early stages of drug development, thus avoiding animal testing and providing relevant *in vitro* toxicity data during pharmaceutical development. Our results point out a higher tolerability to the oil by hepatocytes and epithelial alveolar cells, thus suggesting that for an oral or inhalation administration, higher doses of *L. viridis* L´Hér. essential oil may be used. Taken together the results herein presented show that *L. viridis* L´Hér. essential oils may be a potential natural source of new anti-inflammatory drugs. Nevertheless, *in vivo* studies should be considered to validate the anti-inflammatory potential of this volatile extract.

## 5 Conclusion

The present work highlights the biopotential of an undervalued endemic species, *L. viridis* L´Hér., thus corroborating its traditional uses and concomitantly adding market value and encouraging its industrial exploitation. *L. viridis* L´Hér. showed a relevant essential oil yield and the chemical characterization pointed out a high homogeneity among Portuguese samples, with three main compounds standing out, 1,8-cineole, camphor, and α-pinene. Regarding its biological effects, the volatile extract showed potent antioxidant and anti-inflammatory properties. An inhibition in ROS production as well as inhibitions at transcriptional (*Nos2*, *Il1β* and *Il6*) and in protein (iNOS and COX-2) levels were observed in LPS-stimulated macrophages, culminating in an overall reduction of the inflammatory status. Mechanistically, the inhibitory effects of *L. viridis* L´Hér. essential oil were shown to occur via down-modulation of NF-κB signaling pathway through prevention of IkB-α phosphorylation and a decrease in proteasomal activity. The main compound, 1,8-cineole, seemed to be only partially responsible for the anti-inflammatory properties of *L. viridis* L´Hér. essential oil, suggesting that other compounds may contribute to its activity. Finally, safe concentrations of the volatile extract were disclosed, with oral or inhaled routes of administration being more desirable.

Overall, our results corroborate the traditional uses ascribed to *L. viridis* L´Hér. and open new avenues for the development of plant-based anti-inflammatory agents.

## Data Availability

The original contributions presented in the study are included in the article/Supplementary Material, further inquiries can be directed to the corresponding author/s.
