# Supplementary figures and images for: Lavandula viridis L´Hér. Essential Oil Inhibits the Inflammatory Response in Macrophages Through Blockade of NF-KB Signaling Cascade
Source: Front Pharmacol. 2022 Jan 25;12:695911. doi: 10.3389/fphar.2021.695911 (PMC8821966; doi:10.3389/fphar.2021.695911)

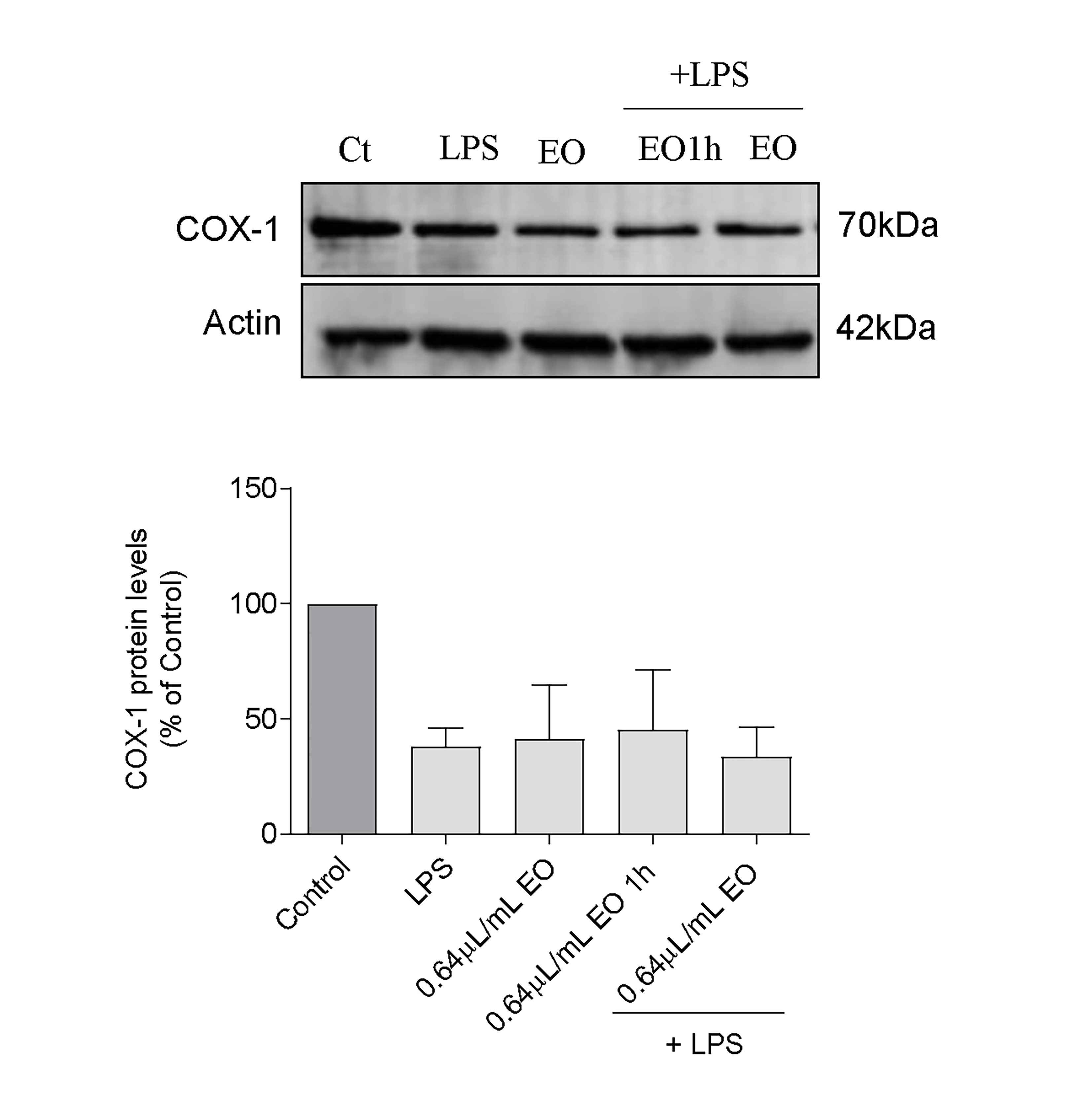

Supplement: Supplementary file 1 [file Image2.jpg]

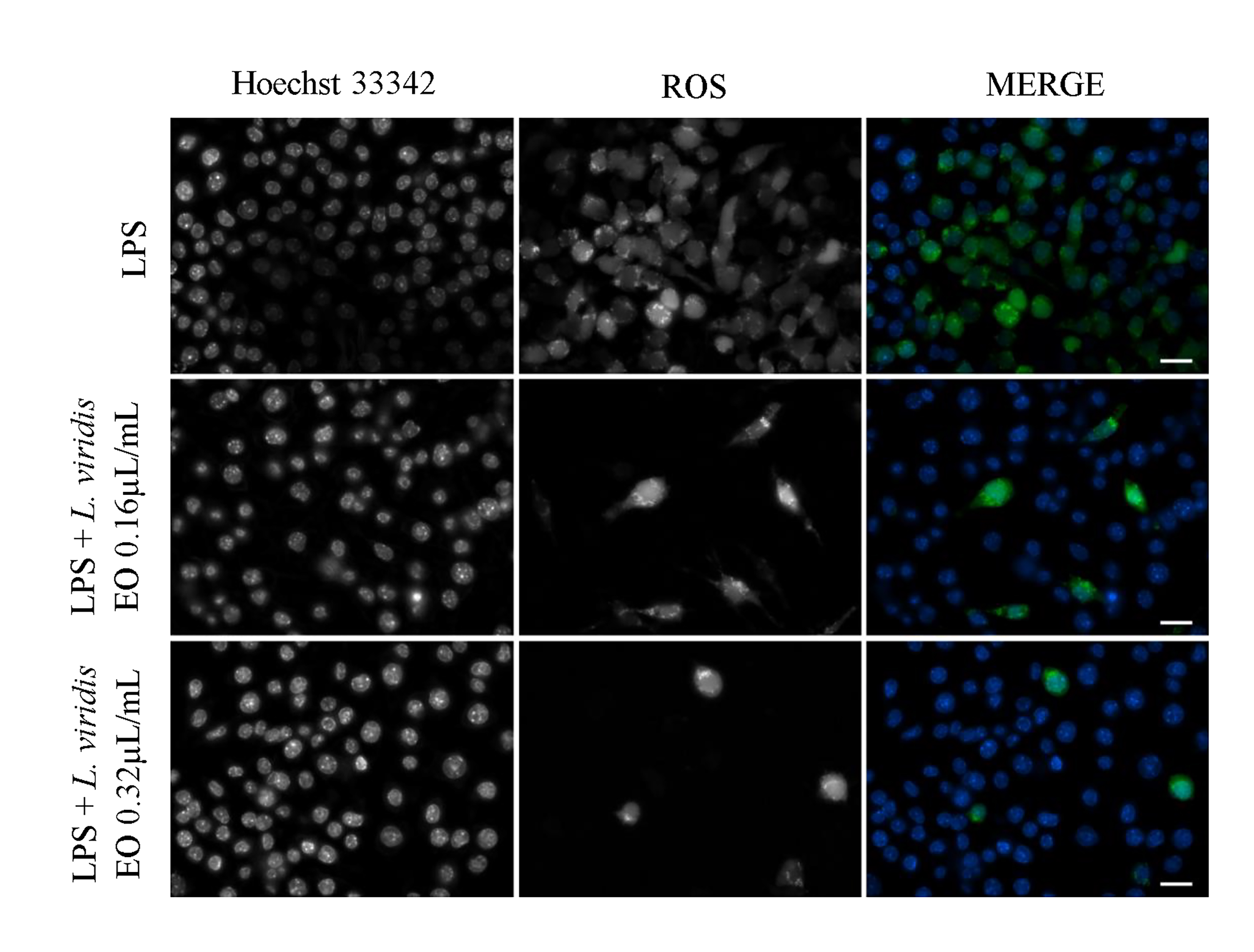

Supplement: Supplementary file 2 [file Image1.jpg]
